# Repeating with the right hemisphere: reduced interactions between phonological and lexical-semantic systems in crossed aphasia?

**DOI:** 10.3389/fnhum.2013.00675

**Published:** 2013-10-18

**Authors:** Irene De-Torres, Guadalupe Dávila, Marcelo L. Berthier, Seán Froudist Walsh, Ignacio Moreno-Torres, Rafael Ruiz-Cruces

**Affiliations:** ^1^Unit of Cognitive Neurology and Aphasia, Centro de Investigaciones, Médico-Sanitarias, University of MálagaMalaga, Spain; ^2^Unit of Physical Medicine and Rehabilitation, Carlos Haya University HospitalMalaga, Spain; ^3^Psychobiology Area, Faculty of Psychology, University of MálagaMalaga, Spain; ^4^Department of Psychosis Studies, Institute of Psychiatry, King's Health PartnersKing's College London, UK; ^5^Department of Spanish Language I, University of MálagaMalaga, Spain

**Keywords:** right hemisphere, language, crossed aphasia, conduction aphasia, language network, structural connectivity

## Abstract

Knowledge on the patterns of repetition amongst individuals who develop language deficits in association with right hemisphere lesions (crossed aphasia) is very limited. Available data indicate that repetition in some crossed aphasics experiencing phonological processing deficits is not heavily influenced by lexical-semantic variables (lexicality, imageability, and frequency) as is regularly reported in phonologically-impaired cases with left hemisphere damage. Moreover, in view of the fact that crossed aphasia is rare, information on the role of right cortical areas and white matter tracts underpinning language repetition deficits is scarce. In this study, repetition performance was assessed in two patients with crossed conduction aphasia and striatal/capsular vascular lesions encompassing the right arcuate fasciculus (AF) and inferior frontal-occipital fasciculus (IFOF), the temporal stem and the white matter underneath the supramarginal gyrus. Both patients showed lexicality effects repeating better words than non-words, but manipulation of other lexical-semantic variables exerted less influence on repetition performance. Imageability and frequency effects, production of meaning-based paraphrases during sentence repetition, or better performance on repeating novel sentences than overlearned clichés were hardly ever observed in these two patients. In one patient, diffusion tensor imaging disclosed damage to the right long direct segment of the AF and IFOF with relative sparing of the anterior indirect and posterior segments of the AF, together with fully developed left perisylvian white matter pathways. These findings suggest that striatal/capsular lesions extending into the right AF and IFOF in some individuals with right hemisphere language dominance are associated with atypical repetition patterns which might reflect reduced interactions between phonological and lexical-semantic processes.

## Introduction

It is well-established that the majority (95%) of right-handers have their left cerebral hemispheres dominant for language (Annett, 1998; Wada and Rasmussen, 2007). A minority (5%) of right-handers have right hemispheric specialization for language (Loring et al., 1990; Annett, 1998; Pujol et al., 1999; Knecht et al., 2002) and mixed language dominance (language production and reception represented in different hemispheres) which can occur in both normal (Lidzba et al., 2011) and brain damaged right-handers (Kurthen et al., 1992; Paparounas et al., 2002; Kamada et al., 2007; Lee et al., 2008) is even more infrequent. The rarity of complete or incomplete lateralization of language to the right hemisphere explains why only a minority of right-handed individuals develop language deficits after right hemisphere injury (crossed aphasia) (Bramwell, 1899; Alexander et al., 1989a; Mariën et al., 2001, 2004). Although crossed aphasia is rare, analysis of language functioning in these subjects represents an ideal opportunity to examine whether their language performance and neural architecture underpinning language functions in the right hemisphere are the same as those reported in subjects with left hemisphere language dominance (Catani et al., 2007; Turken and Dronkers, 2011; Catani and Thiebaut de Schotten, 2012). Here, we report the occurrence of fluent aphasia with severely abnormal repetition and deficits in sentence comprehension (conduction aphasia, CA) in two patients who suffered large right subcortical stroke lesions. This clinical-anatomical correlation is uncommon, but its description can further illuminate the neural organization of propositional language in the right hemisphere. In an attempt to accomplish this, in the present study the localization of damage to white matter tracts underpinning language repetition was outlined in one patient with the aid of brain sections depicted in an atlas of human brain connections (Catani and Thiebaut de Schotten, 2012) and in the other patient with diffusion tensor imaging (DTI) of bilateral white matter tracts.

Knowledge on the organization of propositional language in the right hemisphere comes from the analysis of aphasic patients with damage to the right hemisphere (see Alexander et al., 1989a; Mariën et al., 2004) and from a case series study of intraoperative cortical-subcortical stimulation (Vassal et al., 2010). Vassal coworkers (2010) performed intraoperative cortical-subcortical electrical functional mapping in three right-handed adults who had right-sided low-grade gliomas. Right hemisphere language dominance was variously demonstrated by identification of language deficits during both partial epileptic seizures and preoperative formal testing, and activations in functional magnetic resonance imaging (fMRI) (one patient). During surgical interventions reproducible language disturbances were found by stimulating cortical sites in frontal and temporal cortices. Electrostimulation of the inferior fronto-occipital fasciculus (IFOF) elicited semantic paraphasias, whereas stimulation of the arcuate fasciculus (AF) caused phonemic errors, thus supporting in these cases the hypothesis of a mirror organization of white matter tracts between the right and left hemispheres (Vassal et al., 2010).

Studying patients with crossed aphasia Alexander and colleagues defined two clinical-radiological correlations which were named “mirror image” and “anomalous” (Alexander et al., 1989a; Alexander and Annett, 1996; Alexander, 1997; Mariën et al., 2004). The “mirror image” pattern assumes that the right language cortex has a similar structure and connections to the classical left language cortex, and therefore, similar language deficits to the ones observed after left hemisphere injury can be expected when the same injury occurs in homologous areas of the right hemisphere (Henderson, 1983; Bartha et al., 2004). This pattern occurs in as many as 60% patients and all clinical types of aphasia have been described (see Mariën et al., 2001, 2004). By contrast, the “anomalous” pattern considers that the structural arrangements and functional organization of the language cortex in the right hemisphere are different to the ones in the left language cortex, so that atypical language deficits can occur after right hemisphere injury (e.g., Wernicke's aphasia associated with frontal damage). The anomalous pattern has been described in approximately 40% of patients and it can be easily identified when patients present with relatively isolated phonological or lexical-semantic deficits associated with large lesions in the right perisylvian area (Alexander et al., 1989a; Mariën et al., 2001, 2004). Interestingly, the association of CA with an atypical location is more commonly encountered with right hemisphere lesions (35%) than after left hemisphere involvement (13%) (Basso et al., 1985; Alexander et al., 1989a; Dewarrat et al., 2009). Despite the relatively frequent occurrence of CA in cases of both “mirror image” (Henderson, 1983; Bartha et al., 2004) and “anomalous” crossed aphasia (Alexander et al., 1989a) comprehensive analyses of its main deficits (repetition, short-term memory, sentence comprehension) have been described in only three cases (patient ORL, McCarthy and Warrington, 1984; patient EDE, Berndt et al., 1991; and patient JNR, Berthier et al., 2011). Below, a brief summary of the main findings from patient EDE are described. A further description of the other two cases is not provided here because their personal and developmental histories (mixed handedness and perinatal left hemisphere injury in JNR and left-handedness in ORL) invalidate the diagnosis of crossed aphasia.

Berndt et al. (1991) described the case of a 56-year-old strongly right-handed housewife (EDE) who acutely developed fluent aphasia with impaired auditory comprehension and rapid cycling mood changes in association with a right posterior cortical infarction. A formal evaluation of deficits in EDE was initiated 10 months after the stroke and by that time her reading and writing deficits had improved more than repetition span and auditory sentence comprehension. Since then language and cognitive deficits remained stable and were longitudinally evaluated during the next 5 years. An MRI performed approximately 4 years post-onset revealed a right temporal-parietal infarction compromising cortical regions (middle temporal gyrus and posterior superior temporal gyrus, temporal pole, and posterior insula) engaged in auditory comprehension. In retrospect, it could be argued that EDE probably had an acute Wernicke's aphasia which gradually resolved to CA in the chronic period (1 year post-onset) (Berndt et al., 1991). Berndt and colleagues interpreted the clinical-anatomical relationships observed in EDE as indicative of “mirror image” crossed CA (Alexander et al., 1989a; Alexander and Annett, 1996; Alexander, 1997), although her performance in repetition and short-term memory tasks was atypical in comparison with other patients presenting with short-term memory deficits after left hemisphere damage. Indeed, EDE had intact input phonological processing, 1-item recency effect on list repetition, and absent meaning-based paraphrases during sentence repetition that in the authors' view reflected an atypical interaction between the right and left hemispheres (Berndt et al., 1991). Berndt and her colleagues concluded that in EDE:

*“….there appears to be an unusual dissociation of functions such that the perception of auditory/phonetic information is separated from its storage, while access to semantic information from phonemic forms in connected speech is impaired …… some initial processing of auditory/phonetic information is carried out in EDE's intact left hemisphere, while language functions responsible for phonetic storage and lexical/semantic assignment to sentence constituents are lateralized to the right hemisphere”* (p. 277).

Analysis of repetition performance in the other two patients yielded mixed results. Evaluation in patient JNR replicated the results obtained in EDE (except for abnormal phonological input processing), but patient ORL had repetition deficits similar to the ones described in cases with CA and left hemisphere involvement (see further details in Berthier et al., 2011; McCarthy and Warrington, 1984). In light of the limited data available and mixed results on the pattern of repetition in patients with crossed CA, analysis of further cases is clearly needed. In this study, we specifically investigated repetition deficits in two chronic stroke patients with crossed subcortical CA. We also examined for the first time the role of right white matter pathways involvement in repetition processes in crossed aphasia. Our results replicate findings from previous similar cases (Berndt et al., 1991; Berthier et al., 2011) showing that repetition deficits have atypical features in more demanding tasks (sentence repetition) reflecting limited reliance on lexical-semantic processing as has been reported in typical CA associated to left hemisphere damage. Further, our neuroimaging findings suggest that subcortical lesions in the right hemisphere lesioning perisylvian and commissural pathways may account for the observed language deficits by altering the interaction between right and left hemispheres.

## Methods

### Participants

We examined language deficits including repetition performance (digits, words/non-words, lists of word pairs and triplets, sentences and novel sentences/idiomatic clichés) in two monolingual Spanish speaking patients with chronic CA secondary to large right hemisphere stroke lesions. These two patients were the only ones referred to our unit from 1997 to 2011 with crossed subcortical aphasia.

### Patient JAM

JAM was a 46-year-old man who suffered a large intracerebral haemorrhage in the right striatal/capsular region 1 year before referral to our unit. In the acute period, he had a dense left hemiplegia, left hemianopia, left hemisensory loss, and mild left hemispatial neglect. After a short-lived period of global aphasia, language testing revealed fluent jargon aphasia with impaired auditory comprehension which gradually regressed to CA. Reading and writing were severely affected with features of both deep dysgraphia and deep dyslexia. He also had mild dyscalculia but he did not show ideomotor or buccofacial apraxia as reflected by ceiling scores on the apraxia subtest (60/60) of the Western Aphasia Battery (WAB) (Kertesz, 1982). This later finding is at variance to that commonly observed in patients with CA associated to left hemisphere damage (Geschwind, 1965; Benson et al., 1973; Tognola and Vignolo, 1980). At the time of formal language evaluation JAM was fully oriented and showed adequate insight into his deficits. His affect was flat and he tended to be isolated at home. He met diagnostic criteria for major depression as has been reported in patients with left basal ganglia strokes (Starkstein et al., 1988). JAM was strongly right-handed without history of perinatal injury, developmental delay, or familiar left-handedness. On the Edinburgh Handedness Inventory (Oldfield, 1971) his score was +100. During the first 6 months after the stroke, JAM received conventional speech-language therapy^1^ on an individual basis (2 h/week) showing improvement in spontaneous speech and auditory comprehension. No beneficial changes were reported on repetition deficits.

### Patient AFL

AFL was a 63-year-old woman who developed fluent jargon aphasia with severely compromised auditory and written comprehension in association with a large right subcortical stroke. In the acute period, she had a dense left hemiplegia, left hemianopia, left hemisensory loss but not left hemispatial neglect. Apraxia scores on the WAB were only mildly impaired (49/60) most likely due to comprehension deficits with similar performances on pantomime to verbal commands and pantomime imitation, thus ruling out conduction apraxia (Ochipa et al., 1994). By that time, she was fully aware of her aphasic symptoms in spite of her severe jargon speech and comprehension deficits. She experienced despair, crying very frequently and also showing catastrophic reactions when physicians tried to interact with her (Berthier and Starkstein, 1994). She also met diagnostic criteria for major depression as has been reported in patients with left basal ganglia strokes (Starkstein et al., 1988). AFL was strongly right-handed without history of perinatal injury, developmental delay, or familiar left-handedness. On the Edinburgh Handedness Inventory (Oldfield, 1971) her score was +100. Six months after the stroke, AFL began to receive conventional speech-language therapy^1^ on an individual basis (2 h/week) during a 6 month period showing improvement in spontaneous speech and auditory comprehension. No beneficial changes were reported on repetition deficits.

### Imaging

#### Methods

MRIs studies were performed on different scanners. AFL was studied in 1997 using a 1.5-T Signa scanner (General Electric Medical Systems, Milwaukee, WI) equipped with an eight-channel Philips SENSE head coil. Head movements were minimized using head pads and a forehead strip. High-resolution T_1_-weighted structural images of the whole brain were acquired with three dimensional (3D) magnetization prepared rapid acquisition gradient echo (3 D MPRAGE) sequence (acquisition matrix: 250/250 r; field of view: 240 ms; repetition time [TR]: 2250 ms; echo time [TE]: 238 ms; flip angle: 90; turbo field echo (TFE) factor: 100). The MRI study in JAM was performed on a 3-T magnet (Philips Gyroscan Intera, Best, The Netherlands) equipped with an eight-channel Philips SENSE head coil. Head movements were minimized using head pads and a forehead strap. High-resolution T_1_-weighted structural images of the whole brain were acquired with three dimensional (3D) magnetization prepared rapid acquisition gradient echo (3 D MPRAGE) sequence (acquisition matrix: 240/256 r; field of view: 240 ms; repetition time [TR]: 9.9 ms; echo time [TE]: 4.6 ms; flip angle: 8; turbo field echo (TFE) factor: 200; 1 × 1 × 1 mm^3^ resolution). One hundred eighty two contiguous slices, each 1-mm thick, 0 mm slice gap, were acquired. The total acquisition time of the sequence was about 4:24 min. In addition to the 3D MPRAGE, a standard axial T-2 weighted/FLAIR (TR = 11.000 ms; TE = 125/27 ms; 264 × 512 matrix; field of view [FOV] = 230 × 230; 3-mm-thick slices with 1 mm slice gap) was obtained. A Short TI Inversion Recovery (STIR) was used to produce 24, 2.5 mm axial slices (interslice gap = 1 mm; TR = 4718 ms; TE = 80 ms; inversion time = 200 ms; 264 × 512 matrix; FOV = 230 mm; number of excitations = 2). In both patients the anterior commissure (AC) was identified in axial and coronal T_1_-weighted images at the level of the temporal stems (Warren et al., 2009).

#### Results

**Figure 1:**
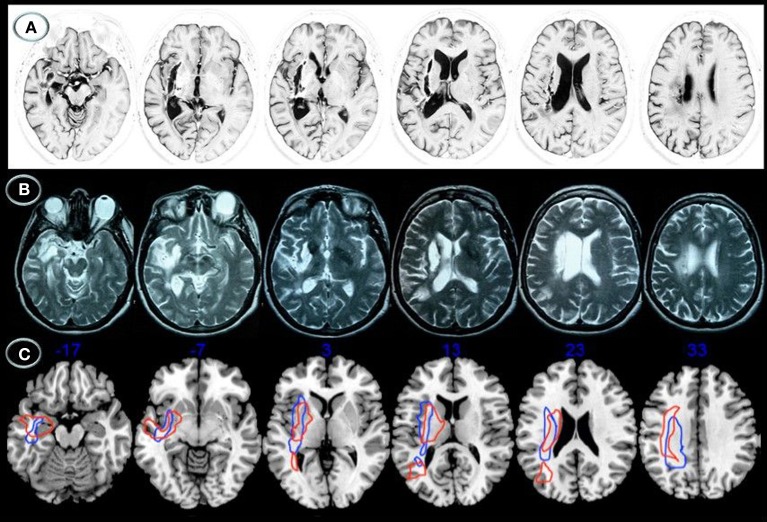
**Structural axial MRI of patients JAM (A) and AFL (B) showing the full extension of lesions**. A 3T MRI (Short T_1_ Inversion Recovery—STIR—sequence) in JAM and 1.5T MRI (T2-weighted sequence) in AFL show relatively similar lesion topographies involving the right striatocapsular region with inferior extension to the temporal stem, ventral insular cortex, and inferior fronto-occipital fasciculus. Note superior extension of the lesions to the arcuate fasciculus and white matter underneath the supramarginal gyrus. Schematic representation of the full extension of lesions **(C)** is depicted in axial MRIcron sections (Rorden, 2005) in JAM (blue lines) and AFL (red lines). The right side is shown on the left.

**Figure 2:**
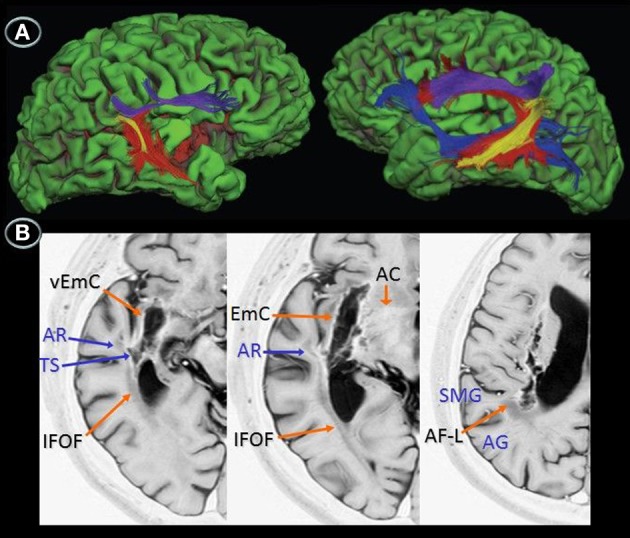
**Diffusion tensor imaging (3T MRI) of patient JAM. (A)** Uninflated surface of the cerebral hemispheres (FreeSurfer reconstruction) depicting gyri in green and sulci in red. The right image shows a small cortical component of the haemorrhage (red) involving the right anterior insula and superior temporal gyrus. The diffusion tensor imaging reconstruction of the arcuate fasciculus and inferior fronto-occipital fasciculus shows (left image) damage to the right long direct segment of the arcuate fasciculus (red) and inferior fronto-occipital fasciculus (blue) with relative sparing of short and long fibers of the anterior indirect segment (purple) and posterior segments (yellow), whereas the right image shows fully developed left perisylvian white matter pathways. **(B)** Anatomical axial MRI section (Short T1 Inversion Recovery—STIR—sequence) show the right striatocapsular lesion and perinecrotic tissue with degeneration of several white matter tracts (orange and blue arrows). AR indicates, auditory radiations; TS, temporal stem; SMG, supramarginal gyrus; AG, angular gyrus; EmC, extreme capsulae; vEmC, extreme capsulae; IFOF, inferior fronto-occipital fasciculus; AC, anterior commissure; AF-L, arcuate fasciculus-long segment.

Lesion location was relatively similar in both patients (Figure 1). Axial MRI showed right basal ganglia lesions including the putamen, part of the external pallidum, and anterior limb, genu, and posterior limbs of the internal capsulae extending superiorly to the periventricular white matter (corona radiata). Tissue damage was also present in the white matter surrounding the hippocampus and the middle temporal gyrus with posterior extension to the auditory and optic radiations in the temporal stem (Figure 2). The right posterior ventral and dorsal insular cortices and the periventricular white matter deep to the supramarginal gyrus were also damaged in both cases, but AFL showed more extensive involvement. No lesions were documented in the left hemisphere.

### Diffusion tensor imaging (DTI)

DTI allows for “*in vivo*” measurement of the diffusive properties of water in a way that allows information to be garnered about the microstructural organization of tissue (Basser et al., 1994). Tractography enables the orientation of white matter (WM) to be ascertained, thus making possible the segregation of WM into separate sections based on the paths of the distinct tracts (LeBihan, 2003). Data acquisition was performed using multi-slice single-shot spin-echo echo-planar imaging (EPI) with specific parameters as follows: FOV 224 mm, 2-mm-thick slices with 0 mm slice gap, *TE* = 117 ms, *TR* = 12408 ms, and b factor: 3000 s/mm^2^. The EPI echo train length consisted of 59 actual echoes reconstructed in a 112 × 128 image matrix. Sixty four diffusion directions were used in order to allow for precise construction of the diffusion tensor. Motion and eddy current correction were performed using FSL's FDT (http://www.fmrib.ox.ac.uk/fsl/) eddy current correction tool (Smith et al., 2004; Woolrich et al., 2009). Diffusion tensor estimation was carried out in using Diffusion Toolkit's least-square estimation algorithm for each voxel (Ruopeng Wang, Van J. Wedeen, TrackVis.org, Martinos Center for Biomedical Imaging, Massachusetts General Hospital). The whole brain tractography used an angular threshold of 35 degrees and an FA threshold of 0.2. The tensor was spectrally decomposed in order to obtain its eigenvalues and eigenvectors. The fiber direction is assumed to correspond to the principal eigenvector (the eigenvector with the largest eigenvalue). This vector was color coded (green for anterior-posterior, blue for superior-inferior and red for left-right) in order to help generate the color FA map. An FA map was also generated from these eigenvalues. This too was done using Diffusion Toolkit. Virtual dissections of the 3 parts of the AF and the IFOF were performed by using a region of interest (ROI) approach, following the directions of a white matter tractography atlas (Catani and Thiebaut de Schotten, 2012). All virtual dissections were performed using TrackVis (Ruopeng Wang, and Van J. Wedeen, TrackVis.org, Martinos Center for Biomedical Imaging, Massachusetts General Hospital).

#### Results

DTI was performed in patient JAM (Figure 2). DTI showed damage to the right long direct segment of the AF and IFOF with relative sparing of the anterior indirect and posterior segments of the AF together with fully developed left AF and IFOF. Since DTI could not be performed in patient AFL the white matter tracts affected by the lesion were identified with the aid of an atlas of human brain connections (Catani and Thiebaut de Schotten, 2012). The outline of white matter tracts in patient AFL suggested that both right AF and IFOF were damaged.

### Language assessment

While both patients had Wernicke's aphasia in the subacute period, language deficits were more severe in AFL than in JAM. This was also reflected in the chronic period by the scores obtained in the Western Aphasia Battery (WAB) (Kertesz, 1982); JAM had an Aphasia Quotient of 79.6 (mild to moderate aphasia) and AFL of 56.4 (moderate to severe aphasia). JAM showed a combination of fluent and well-articulated spontaneous speech with rare phonemic paraphasias and occasional approximation to target words to repair errors (*conduite d'approche*), preserved auditory comprehension except for sequential commands and impaired repetition of multisyllabic words and sentences. Naming was relatively preserved. His WAB scores (fluency: 9, comprehension: 7.4, repetition: 6.2, naming: 9.2) were consistent with the diagnosis of CA (Kertesz, 1982). AFL showed fluent and well-articulated speech with mixing fragments of phonemic jargon and occasional normal utterances. Comprehension of sequential commands, sentence repetition, and naming were moderately impaired. Her WAB scores (fluency: 7, comprehension: 6.6, repetition: 5.7, naming: 3.9) were consistent with the diagnosis of CA (Kertesz, 1982).

### Experimental assessments

To explore the interaction between phonology and lexical-semantic processing, both patients were evaluated using selected subtests from the Psycholinguistic Assessments of Language Processing in Aphasia (PALPA) (Kay et al., 1992; Valle and Cuetos, 1995; Kay and Terry, 2004) and a battery of experimental tests (Berthier, 2001).

### Phonological processing

#### Word pair discrimination

***Method.*** Four PALPA subtests were used to evaluate auditory processing for discriminating minimal pairs. These included Non-word Minimal Pairs (PALPA 1), Word Minimal Pairs (PALPA 2), Word Minimal Pairs Requiring Written Selection (PALPA 3), and Word Minimal Pairs Requiring Picture Selection (PALPA 4). The minimal pairs tests from the PALPA required same/different judgments for pairs of monosyllabic words/non-words that differed by a single phonetic feature (e.g., “sol-col” [sun-cabbage]). In half the trials, the two stimuli were identical and in half they were different.

***Results.*** Both patients had abnormal performance on auditory discrimination of non-word minimal pairs with relatively similar scores on same and different pairs in AFL and significantly better performance on same pairs in JAM relative to different pairs which resulted from his tendency to classify most pairs as similar [χ^2^_(1)_ = 25.2, *p* < 0.0001]. Performance was significantly better discriminating identical minimal word pairs than different word pairs in both JAM [χ^2^_(1)_ = 9.68, *p* = 0.002] and AFL [χ^2^_(1)_ = 9.24, *p* = 0.002]. AFL had impaired performance in auditory discrimination of word minimal pairs requiring written selection (this test was not administered to JAM). Scores in word minimal pairs requiring picture selection were relatively preserved in JAM and AFL Table 1.

**Table 1:**
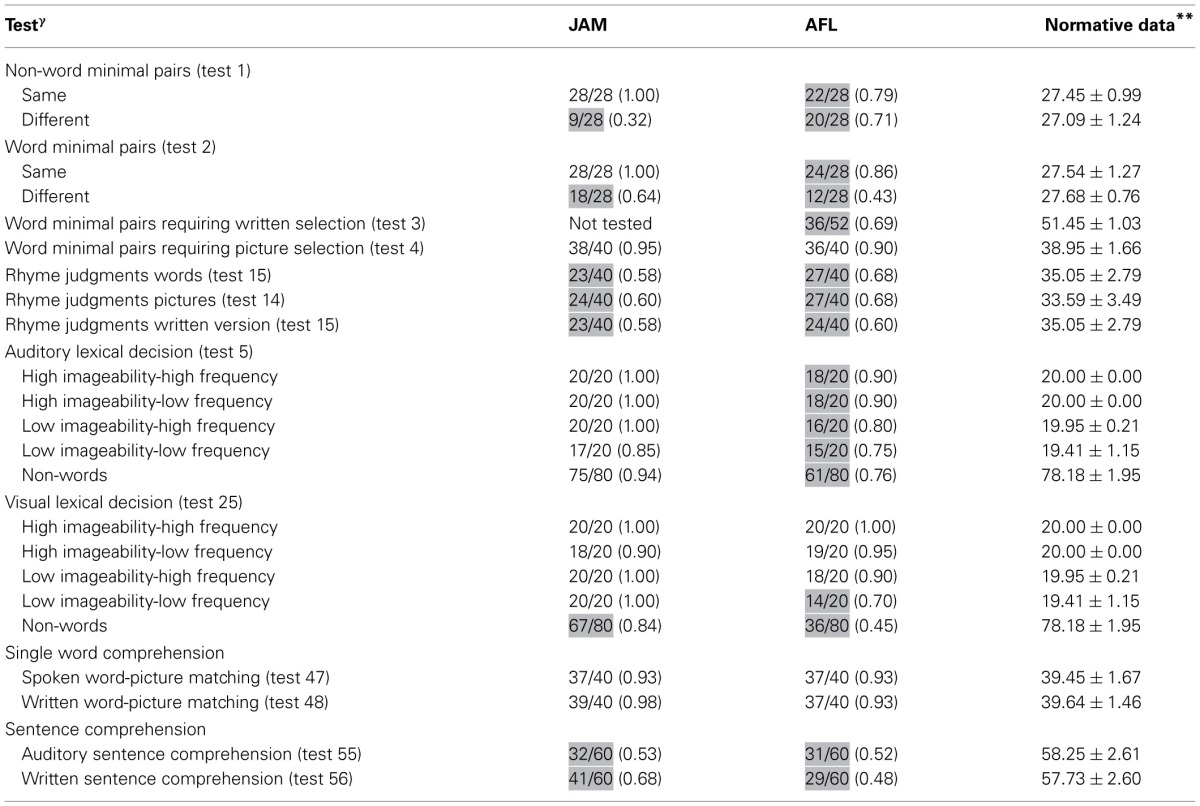
**Auditory and reading processing**.

^γ^Test number follows the nomenclature of the original English version of PALPA (see Kay and Terry, 2004) which is slightly different from the Spanish version. Numbers in parentheses indicate proportion of correct responses. ^**^Normative data from Valle and Cuetos (1995). Abnormal results are highlighted in gray. See further information in text.

### Rhyme judgments

#### Method

Three PALPA subtests were used to evaluate processing for Rhyme Judgments in Auditory/Written (PALPA 15) and Pictures (PALPA 14) presentations. In each rhyme judgment task, two words were presented in the corresponding modality and the patient was required to say whether or not they rhymed (e.g., “tarta-carta” [cake-letter]). There were 40 trials divided equally between rhyming and non-rhyming pairs.

#### Results

The ability of both patients to make rhyme judgments was abnormal in all modalities of presentation (auditory and written words and pictures) (Table 1).

### Lexical processing

#### Lexical decision

***Methods***. Word/non-word discrimination was assessed with the Auditory Lexical Decision: Imageability × Frequency (PALPA 5) and the Visual Lexical Decision: Imageability and Frequency (PALPA 25). These two versions were administered 2 weeks apart to prevent learning. These tests use 80 words of high- and low-imagery and high- and low- frequency and 80 non-words derived from each of the real words by changing one or more letters. All non-words follow Spanish spelling rules and were pronounceable (Valle and Cuetos, 1995).

***Results.*** JAM performance on Auditory Lexical Decision was preserved for words (77/80) and non-words (75/80) [χ^2^_(1)_ = 0.13, *p* = 0.718]. Misses occurred in three low-imageability/low-frequency items (“anger,” “dogma,” “satire”), whereas false alarms in non-words were derived from low-imageability words (Table 1). Although AFL's performance in this task was abnormal, she recognized words (67/80) and non-words (61/80) with similar efficiency [χ^2^_(1)_ = 0.97, *p* = 0.325]. Most of her misses (e.g., “irony,” “method,” “satire”) and false alarms occurred in low-imageability words and in non-words derived from low-imageability words. On Visual Lexical Decision JAM had better recognition of words (78/80) than non-words (67/80) [χ^2^_(1)_ = 7.31, *p* = 0.007]. A similar dissociation was found in AFL (words = 71/80; non-words = 36/80) [χ^2^_(1)_ = 32.4, *p* = 0.0001].

### Single word comprehension

#### Method

Single word comprehension was assessed with the Spoken Word—Picture Matching (PALPA 47) and the Written Word—Picture Matching (PALPA 48) tasks. The two versions were administered 2 weeks apart to prevent learning. These tasks required that the patient match a spoken or a written word to one of five pictures (target nouns and four distractor items [one closely semantic, one distantly semantic; one visual, and one unrelated]).

#### Results

The performance of both patients was relatively preserved on the auditory and written presentations (Table 1).

### Sentence comprehension

#### Method

Sentence comprehension was assessed using the Auditory Sentence Comprehension (PALPA 55) and the Written Sentence Comprehension (PALPA 56) tasks. These two versions were administered 2 weeks apart to prevent learning. These tasks require matching an auditory or written sentence presented with one of three figures, the target one and two distractors. Several types of sentences were examined including reversible (e.g., “The dog is approaching the girl”) and non-reversible (e.g., “The dog is washed by the girl”) sentences, active and passive sentences, directional and non-directional sentences, and gapped sentences.

#### Results

Both patients showed severely impaired performance in both auditory and written modalities of presentation. Their performance was similar for reversible and non-reversible sentences (Table 1).

### Digit production and matching span

#### Method

This was assessed with the Digit Production/Matching Span (PALPA 13).

#### Results

Both patients has restricted digit production and matching span (Table 2).

**Table 2:**
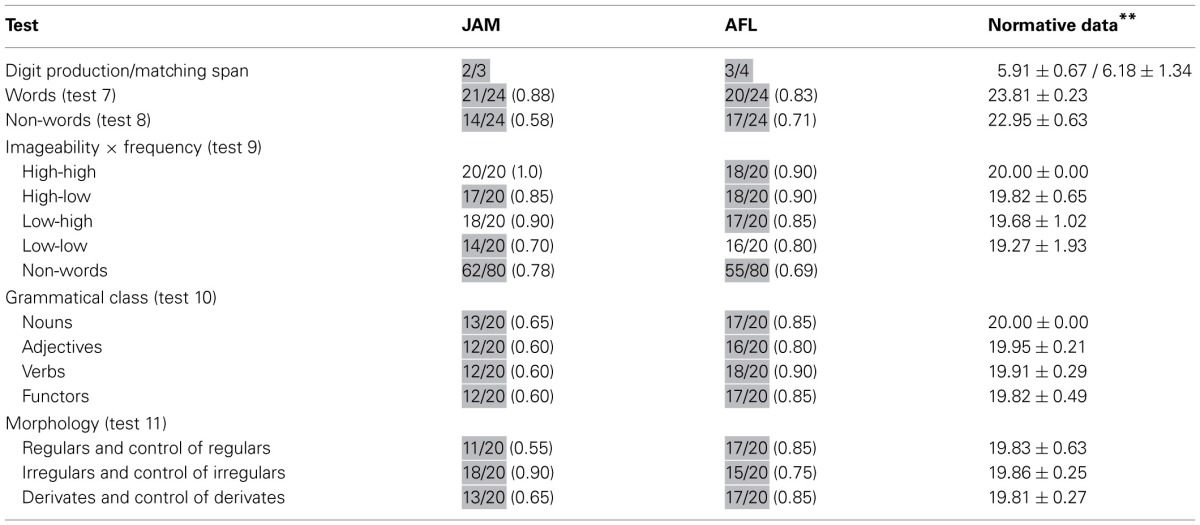
**Auditory processing: repetition of digits, single words, and non-words**.

Numbers in parentheses indicate proportion of correct responses.^**^Normative data from Valle and Cuetos (1995). Abnormal results are highlighted in gray. See further details in text.

### Repetition of words and non-words

#### Method

Length, frequency, and imageability of words can influence the accuracy of repetition amongst aphasic patients. Studies in CA suggest that repetition of short words is better than repetition of multisyllabic and grammatical words (Goodglass, 1992; Nadeau, 2001). Therefore, performance on output phonological tasks was assessed with two repetition subtests [Repetition: Syllable Length (PALPA 7) and Repetition: Non-words (PALPA 8)]. These tests contain 24 words and 24 non-words of increased length (3–6 letters). To further evaluate potential dissociations in repetition performance between words and non-words, the Repetition: Imageability × Frequency (PALPA 9) subtest was also administered. This test contains 80 words and 80 non-words presented in a mixed fashion. Words were grouped in four lists (20 items in each list) with variations in frequency and imageability. The lists contained high-frequency/high-imageability, high-frequency/low-imageability, low-frequency/high-imageability, and low-frequency/low-imageability words. These lists were matched for syllable length; items contained between one and four syllables. The non-words were matched to the words for phonological complexity. Errors in all repetition tasks were analyzed by two of us (ID-T, GD).

#### Results

Word repetition (PALPA 7) was mildly impaired in JAM (0.88) and AFL (0.83). Scores in word repetition were marginally better than those found in non-words (PALPA 8) in JAM [χ^2^_(1)_ = 3.72, *p* < 0.054], whereas similar performances were found in AFL [χ^2^_(1)_ = 0.46, *p* = 0.494] (Table 2). In PALPA 9, no differences were found in JAM [χ^2^_(1)_ = 1.51, *p* = 0.22], but AFL repeated words significantly better than non-words [χ^2^_(1)_ = 6.02, *p* = 0.014]. Regarding word repetition in PALPA 9 test, both patients repeated items of the four lists with relatively similar efficiency. Repetition of low-imageability and low- frequency words in JAM (0.70) and AFL (0.80) was slightly poorer than repetition of the other lists, but differences did not reach significance. It should be noted that most non-words in the Spanish version of the PALPA 9 (Valle and Cuetos, 1995) have high word-likeness (Gathercole and Marin, 1996) because they are derived from words with a single consonant (*n* = 30; “pierna” [leg] → *pierla*) or a vowel [*n* = 22; “hospital” (hospital) → *hospitel*] exchanged. While word-likeness increases the likelihood of lexicalization on repetition tasks in patients with typical CA and left hemisphere damage (Saito et al., 2003), this was not the case in our patients as lexicalizations during non-word repetition (PALPA 9) were rare (JAM: 4/80 [0.05]; AFL: 5/80 [0.06]).

### Repetition: grammatical class and morphology

#### Method

Grammatical class (PALPA 10) and morphological endings (PALPA 11) were evaluated in both patients. PALPA 10 evaluates the effect of grammatical class. This test contains 80 words grouped in four different categories (nouns, adjectives, verbs, and functors) of 20 items in each list. PALPA 11 evaluates whether repetition is affected by morphological endings. This test contains 60 words grouped in three lists (regulars and control of regulars, irregulars and control of irregulars and derivates and control of derivates).

#### Results

Scores in PALPA 10 ranged from mildly (0.80) to moderately (0.60) impaired in AFL and JAM, but repetition performance was not influenced by grammatical class. Repetition with different morphological endings was mildly impaired in AFL, but her performance was relatively similar regardless the type of morphological endings. JAM had low average (0.90) repetition of irregulars and controls of irregulars and moderately impaired (0.60) regular and derivates and their controls (Table 2).

### Word pair repetition

#### Method

To assess the influence of lexical-semantic information on repetition ability when the demand of the auditory-verbal short-term memory is increased both patients were asked to repeat word pairs (e.g., “house-flower”) (*n* = 56). Patients were asked to repeat immediately after auditory presentation in a no-delay direct condition (Martin et al., 1996; Gold and Kertesz, 2001) a total of 112 high-frequency words. The total list was composed of high-frequency/high imageability (*n* = 28), high-frequency/low-imageability (*n* = 28); low-frequency/high-imageability (*n* = 28) and low-frequency/low-imageability (*n* = 28) words. Responses were scored for the number of word pairs repeated verbatim and for the number of words repeated accurately as a function of serial position (initial and final) in the list, irrespective of whether the word pair was repeated accurately or not. The number of correct words, failures to respond, and semantic, phonologic, formal, neologistic, perseverative, and unrelated lexical errors was evaluated.

#### Results

Performance on this task was moderately impaired in both patients. Table 3 shows the number of word pairs that were repeated correctly. Further analyses disclosed that JAM repeated correctly 74 of the total 112 (0.66) words. There was a serial position effect (initial = 43/56; terminal = 26/56) [χ^2^_(1)_ = 9.58, *p* = 0.002] which may be attributable to his markedly reduced memory span (2 items). There were no effects of frequency/imageability. Abnormal responses were ordered by the frequency of occurrence and included: failures to respond = 17 (0.44), phonological errors = 7 (0.19), neologisms = 5 (0.14), formal errors = 4 (0.11), unrelated errors = 4 (0.11), and perseverations = 1 (0.2). There were no semantic errors. AFL repeated correctly 67 of the total 112 words (Table 3). There was a marginally significant effect of frequency/imageability since she showed better repetition of high-frequency/high imageability word pairs than for high-frequency/low-imageability word pairs [χ^2^_(1)_ = 3.51, *p* < 0.061], but there were no other differences. There were no serial position effects (initial = 30/56; terminal = 29/56) on word pair repetition which may be attributable to her memory span (3 items). Her responses included phonological errors = 22 (0.49), neologisms = 11 (0.24), formal errors = 7 (0.16), failures to respond = 3 (0.07), unrelated errors = 1 (0.02), and perseverations = 1 (0.2). There were no semantic errors.

**Table 3:**
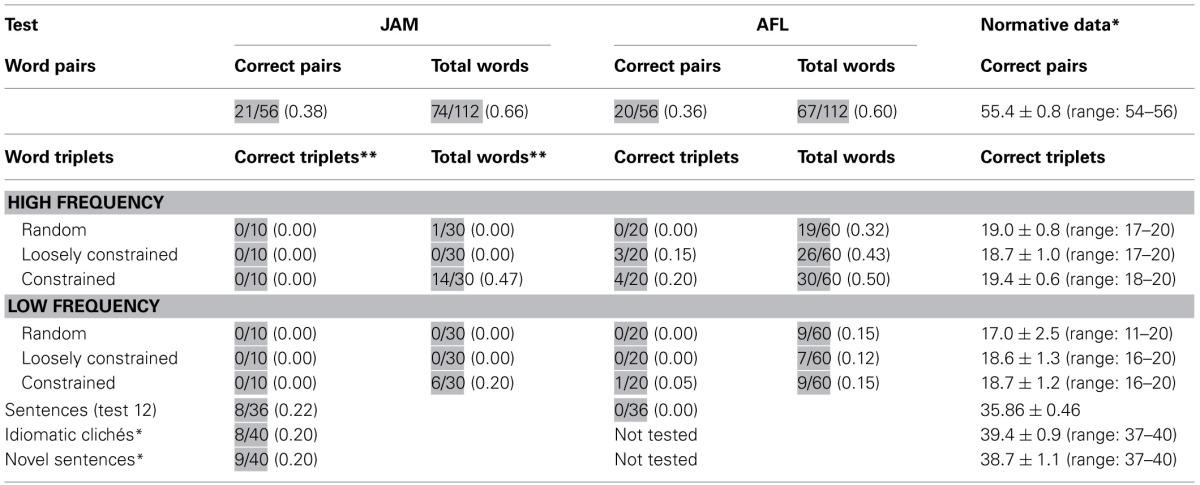
**Auditory processing: repetition of word pairs, word triplets, sentences, and clichés**.

Numbers in parentheses indicate proportion of correct responses. Abnormal results are highlighted in gray. ^*^Taken from Berthier (2001) except test 12. ^**^Note that testing of word triplets in JAM was partially administered. See further details in text.

### Repetition of word triplets

#### Methods

Patients were also asked to repeat word triplets. This task is a modification of the one used by McCarthy and Warrington (1984, 1987) in patients with CA. In the present battery two sets of 60 three-word lists (verb-adjective-noun) were created (Berthier, 2001). These were composed of word strings of increasing semantic richness that is from non-organized to organized semantic information. Two 20 three-word lists (List 1: 60 high-frequency words; List 4: 60 low-frequency words) consisted of random word combinations (e.g., "buy-sweet-country"). Two other 20 three-words lists (List 2: 60 high-frequency words; List 5: 60 low-frequency words) conveyed loosely constrained meaningful information (e.g., "defend-hero-gold”), and two other 20 three-word lists (List 3: 60 high-frequency words; List 6: 60 low-frequency words) conveyed closely constrained meaningful information (e.g., “cut-lovely-flower”). Words were read at a rate of one per second and patients were required to repeat the words in the order given by the examiner. Responses were scored for the number of lists repeated verbatim in each condition and for the number of words repeated accurately as a function of serial position (initial, medial and final) in the list, irrespective of whether the whole triplet was repeated accurately or not. The number of correct words, failures to respond, and semantic, phonologic, formal, neologistic, perseverative, and unrelated lexical errors was evaluated.

#### Results

Performance on this task was severely impaired in both patients (Table 3). JAM failed to repeat any word triplet correctly (e.g., “read-new-book” → *read ….don't know*). Since he became frustrated after repeated unsuccessful attempts the task was discontinued after 10 consecutive failures in each list. Analysis of individual words during these interrupted trials indicated that JAM repeated more words in triplets rich in semantic relations than in the other lists, showing significantly better performances in high-frequency triplets than low-frequency triplets [χ^2^_(1)_ = 4.17, *p* < 0.041]. AFL could only repeat 8 out of 120 (0.07) word triplets correctly (List 2 = 3; List 3 = 4; List 6 = 1) and there was a trend for better repetition of high-frequency than low-frequency word triplets [χ^2^_(1)_ = 3.32, *p* < 0.068]. Analysis of semantic relatedness in high-frequency lists (Lists 1, 2 and 3) showed a trend for better repetition of List 3 (constrained meaningful information) than List 1 (random word combination) [χ^2^_(1)_ = 3.42, *p* < 0.064], but there were no further differences in the other high-frequency lists or in the low-frequency lists. Analyses of performance in repeating individual words disclosed that AFL produced more correct items while repeating high-frequency (75/180 [.42]) than low-frequency (25/180 [.12]) triplets [χ^2^_(1)_ = 33.1, *p* < 0.0001]. For the sake of simplicity, error analysis in both patients was performed considering only the total number of errors regardless the list in which they occurred. Abnormal responses were ordered by the frequency of occurrence. Note that since in JAM this task was interrupted after 10 consecutive failures in each list, only 180 words could be analyzed. His responses were failures to respond = 144 (0.80), semantic errors = 5 (0.03), perseverations = 4 (0.02), phonological errors = 3 (0.02), unrelated errors = 2 (0.01), and neologisms = 1 (0.00). The total number of words (*n* = 360) could be analyzed in AFL and her abnormal responses included phonological errors = 95 (0.36), neologisms = 50 (0.19), perseverations = 43 (0.16), failures to respond = 41 (0.16), formal errors = 13 (0.05), unrelated errors = 12 (0.05) and semantic errors = 6 (0.02). Although their responses contained a number of errors, none of them produced meaning-based paraphrases. Patients' performance according to the serial position in the list were relatively similar for initial (JAM = 0.3; AFL = 0.27), medial (JAM = 0.1; AFL = 0.23), and terminal (JAM = 0.7; AFL = 0.36) positions.

### Repetition of sentences

#### Methods

Sentence repetition was assessed with the PALPA 12. This task evaluates the ability to repeat auditorily-presented sentences (*n* = 36) of different length (from 5 to 9 words). It is composed of reversible sentences (*n* = 20) and non-reversible (*n* = 16) sentences. Serial position curves were generated for all 7-word sentences (*n* = 18).

#### Results

Sentence Repetition (PALPA 12) was severely abnormal in both patients (Table 3). In fact, AFL failed to repeat a single sentence correctly, whereas JAM had less difficulty and could repeat some non-reversible sentences yet his performance was severely abnormal (8/36 [.22]). Error analysis revealed that both patients omitted many words and mainly produced phonological errors; neologisms were also heard in AFL. Semantic paraphasias were not observed in AFL, but JAM produced rare semantic errors (“man” → *owner*) and semantic perseverations. Paraphrases of target sentences were conspicuously absent in AFL. In JAM there were no paraphrases in strict sense, except for the presence of a difficult to classify sentence (sentence 17: “This dog has more cats to chase” → *This dog … this cat, there are more to run*) in which the meaning of the original sentence was not fully replicated in the response (Saffran and Marin, 1975). Analyses of serial position curves of seven word sentences revealed a tendency for repeating initial (items 1 and 2) and terminal (item 6) words (range of correct for these positions: 60–80%) correctly with frequent omissions (range of correct: 20–40%) of words in the midportion of sentences (items 3, 4, 5) in JAM. A more inconsistent pattern was seen in AFL who tended to reproduce more regularly words in certain positions (items 1, 3, 5, 6) (range of correct: 60–80%) than the words in other positions (range of correct: 25%–50%).

### Repetition of clichés and novel sentences.

#### Method

To explore possible dissociation between both types of sentences, JAM was asked to repeat familiar idiomatic Spanish sentences (clichés) (*n* = 40) taken from the 150 Famous Clichés of Spanish Language (Junceda, 1981) as well as a set of novel sentences (*n* = 40) that were construed following the methodology described by Cum and Ellis (1999) and Berthier et al. (2011). For example, for the idiomatic cliché: “Me lo dijo un pajarito” (“A little bird told me”) the novel control sentence: “Me lo dijo mi compadre” (“My friend told me”) was created. This task was not administered to AFL.

#### Results

JAM was moderately impaired in these tasks obtaining relatively similar scores in both types of sentences. He rarely made paraphrases in novel sentence repetition (3/40 [.08]) and only 1 paraphrase (1/40 [.02]) was heard in repetition of idiomatic clichés (“Mess things up” → *Make a mess*).

## Discussion

We have described the profile of language deficits in two chronic aphasic patients. They did poorly in input phonological tasks (minimal pairs, rhyme judgments) when stimuli were presented in auditory and written modalities. Lexical-semantic processing for single words (lexical decision, comprehension) was relatively preserved in these input modalities, but both patients infrequently accessed meaning when asked to comprehend and repeat complex verbal messages. Indeed, a relatively preserved performance in single word repetition contrasted with a severe impairment in repetition of digits, non-words, word lists, sentences, novel phrases and idiomatic clichés. In several instances, repetition was not significantly influenced by the frequency, imageability, and lexicality of stimuli. This atypical combination of language deficits could also be deemed uncommon because they took place in two strongly right-handed patients with residual crossed CA associated with predominantly right striatal/capsular lesions also affecting the AF, IFOF, anterior commissure, and temporal stem. The distinctive features of this clinical-anatomical correlation are discussed below.

### Crossed subcortical aphasia

Crossed subcortical aphasia is a rare condition to the extent that in a recent review of the literature only nine cases met criteria for “possibly reliable” or “reliable” diagnosis (De Witte et al., 2008). During the acute and early chronic periods both JAM and AFL most likely had Wernicke's aphasia and left hemiplegia which resulted from extensive right striatal/capsular lesions extending into the temporal stem/IFOF and supramarginal gyrus/AF. This clinical-anatomical correlation likely represents the right-sided analogue to the syndrome of Wernicke-type aphasia with right hemiparesis secondary to left subcortical injury originally described by Naeser et al. (1982). This syndrome, which is considered a rare entity (Wolfe and Ross, 1987), usually occurring with atypical language deficits (Damasio et al., 1982), has not been well-defined in crossed aphasic patients (Basso et al., 1985). In their original publication, Naeser and colleagues (1982) described three aphasic syndromes associated with left capsular/putaminal involvement and variable lesion extension to either anterior-superior, posterior, or both anterior-superior and posterior neighboring structures. Of these, the syndrome that best fits with the one we found in JAM and AFL after right hemisphere injury is characterized by poor comprehension, fluent Wernicke's type speech, and lasting right hemiplegia in association with left capsular/putaminal damage and posterior lesion extension to the auditory radiations in the temporal stem (Cases 4, 5, and 6 in Naeser et al., 1982, pp. 8–10). In Naeser et al.'s case series (1982) testing in the chronic period was possible in two patients and it revealed improvement in all language modalities in one patient and no changes in the other.

Our patients may be interpreted as presenting “mirror image” crossed CA (Alexander et al., 1989a) for two reasons: (1) similar surface symptoms and lesion topography to the syndrome described after left hemisphere involvement; and (2) gradual resolution of language deficits from receptive aphasia to a less severe CA as is regularly described in cases with Wernicke's aphasia and left hemisphere lesions (Goodglass, 1992). Regrettably, in the aphasic patients with left “capsular/putaminal with posterior lesion extension” described by Naeser et al., 1982 language deficits (including repetition) were succinctly described, thus making it hard to establish whether or not their intrinsic characteristics were typical. Increasing our understanding on this issue is desirable because evaluation of repetition deficits in patients with “mirror image” crossed CA has been performed only in patient EDE who unexpectedly showed atypical performance on word list and sentence repetition (Berndt et al., 1991). This would mean that repetition deficits in some cases with right-hemisphere language dominance deviate from the classical pattern reported in similar cases with left hemisphere dominance because the neural organization of language in the former is different. Regrettably, the scarcity of similar well-studied cases and the reported heterogeneity in demographic and clinical-anatomic variables prevent further elaborations. It suffices to say that atypical neural organization of language in the right hemisphere may apply for patient EDE with right temporal-parietal involvement (Berndt et al., 1991) but possibly not for ORF, a left-handed conduction aphasic patient with right parietal damage and good access to meaning during word list and sentence repetition (McCarthy and Warrington, 1984).

It is even more difficult clarifying the finding of atypical language deficits in our crossed aphasic patients with striatal/capsular involvement because atypical language deficits are common in left subcortical aphasia (Albert et al., 1981; Damasio et al., 1982; Fromm et al., 1985) and because the role of left basal ganglia in language deficits is still controversial (Damasio et al., 1982; Naeser et al., 1982; Cappa et al., 1983; Nadeau and Crosson, 1997). Most studies evaluating subcortical stroke provided evidence against a prominent role of basal ganglia in language and instead attributed language deficits to the deleterious effect of subcortical involvement on the overlying cortex (Nadeau and Crosson, 1997; Hillis et al., 2002; Radanovic and Scaff, 2003; de Boissezon et al., 2005; Choi et al., 2007). One study on vascular aphasia secondary to left subcortical lesions mainly affecting the striatum ascribed lexical-semantic deficits to dysfunction of the basal temporal language area and IFOF (de Boissezon et al., 2005). Anatomical data in our patients with crossed CA also suggest that the pattern of language deficits (impaired sentence comprehension, sentence repetition) may be linked to damage to the right basal temporal language area and white matter tracts rather than to the striatocapsular lesions.

### Dissociated structure-function relationships in crossed subcortical aphasia?

There is some evidence that the AF is asymmetric being larger in volume and having a higher fiber density in the left hemisphere compared to the right (Parker et al., 2005; Powell et al., 2006; Vernooij et al., 2007; Catani and Mesulam, 2008; Axer et al., 2012; Catani and Thiebaut de Schotten, 2012). Combining DTI and fMRI in a small group of strongly right-handed healthy subjects, Powell et al. (2006) demonstrated for the first time that a greater development of left hemisphere white matter tracts in comparison with their homologues counterparts correlated with left-sided lateralization of language function. Although this structure-function correspondence has been replicated in subsequent studies (Matsumoto et al., 2008; Saur et al., 2008), other studies variously combining DTI with fMRI, Wada test, or other ancillary methods (resting-state functional connectivity analysis) have questioned the long-held assumption that leftward asymmetry in volume of cortical areas (planum temporale) and white matter pathways underlie functional lateralization (see references in Vernooij et al., 2007; Turken and Dronkers, 2011). In complimentary terms, differences in the intra- and inter-hemispheric architecture and function of perisylvian white matter tracts exist and might account for the distinct performance in verbal repetition in healthy subjects (Catani et al., 2007) and in patients presenting with contrasting aphasic deficits (conduction aphasia *versus* transcortical aphasias) (Catani et al., 2005; Berthier et al., 2012). In fact, DTI studies reveal intra- and inter-hemispheric variability of white matter pathways underpinning repetition, most notably of the AF/superior longitudinal fasciculus (SLF) (Nucifora et al., 2005; Catani and Mesulam, 2008; Gharabaghi et al., 2009; Friederici and Gierhan, 2013). Leftward biased asymmetry of the AF/SLF predominates in males and usually coexists with the absence or vestigial development of its long segment in the right hemisphere (Catani et al., 2005; Powell et al., 2006; Catani and Mesulam, 2008; Thiebaut de Schotten et al., 2011; Catani and Thiebaut de Schotten, 2012; Häberling et al., 2013) although at least one study reproduced the left hemisphere architecture and connectivity in the right hemisphere (Gharabaghi et al., 2009). Another study found reversed asymmetry of the AF in healthy males with right hemisphere language lateralization (Häberling et al., 2013). More symmetric patterns (bilateral-left and bilateral) of the AF/SLF prevail in females (~40%) and some researchers consider that other white matter bundles (IFOF) are also less lateralized than the dorsal stream but this has not been confirmed in all studies (Cao et al., 2003; Rodrigo et al., 2007). Regarding function of the AF/SLF, recent studies using Wada test (Matsumoto et al., 2008) or fMRI (Saur et al., 2008) documented leftward lateralization in subjects with left hemisphere dominance for language; however, it has also been shown that left-handers with right hemisphere language dominance (as seen using fMRI) (Vernooij et al., 2007) actually have left-lateralized AF. Taken together these later findings align with the hypothesis that lateralized hemispheric function is not always guided by structural asymmetry (Wada, 2009). In support of this view, we did find dissociation between structure and function in JAM. The extensive right subcortical lesion in JAM hindered not only the comparison of inter-hemispheric AF and IFOF architecture but also the possibility of ruling out a reversal of the anatomical asymmetry. Nevertheless, the DTI identified well-developed residual components (anterior indirect and posterior segments) of the right AF/SLF that have escaped from tissue damage together with fully developed AF and IFOF in the left hemisphere which suggest symmetric or leftward lateralization. Despite this structural arrangement, JAM had right hemisphere dominance for language as reflected by his severe and long-lasting repetition disorder consequential to damage to the right AF/SLF and IFOF. Our study did not provide direct evidence of the functional activity of the left white matter tracts (AF, IFOF), yet the persistence of severe deficits on repeating (non-words, word lists and sentences) and accessing meaning during both sentence comprehension and repetition 1 year after stroke onset makes the natural and therapy-based compensation of such deficits by means of the fully-developed left white matter tracts negligible. Nevertheless, further studies are clearly needed to establish the structure-function relationships amongst individuals with atypical language lateralization.

### Is repetition atypical in crossed subcortical aphasia?

In both JAM and AFL word repetition scores ranged from normal to mild impairment but their performance in non-word repetition was markedly abnormal, a profile generally described in patients with CA and left hemisphere damage (Caplan and Waters, 1992; Goodglass, 1992). Functional neuroimaging in healthy subjects shows activation of superior temporal and premotor cortices bilaterally during single word repetition, whereas non-word repetition activates the same cortical regions mostly in the left hemisphere (Weiller et al., 1995; Saur et al., 2008). Studies combining fMRI with DTI reveal interaction between superior temporal and premotor areas during sublexical repetition via the AF/SLF (Saur et al., 2008). Based on these observations the likely mechanism accounting for the superior performance in JAM and AFL on repeating words over non-words may be the conjoint activity of residual areas of the injured right hemisphere and the intact left hemisphere (Weiller et al., 1995; Ohyama et al., 1996; Abo et al., 2004). Poor non-word repetition may be the expected consequence of right hemisphere damage with limited possibility of natural left hemisphere compensation. In support, lesion analysis in both patients and DTI findings in JAM showed massive involvement of the long direct segment of the AF normally engaged in auditory/phonological transcoding (word and non-word repetition) (Catani et al., 2005; Saur et al., 2008; Catani and Thiebaut de Schotten, 2012; Cloutman, 2012; Friederici and Gierhan, 2013). It should be noted, however, that their performance in other repetition tasks differed in a number of important respects from typical CA associated with left hemisphere lesions (Saffran and Marin, 1975; McCarthy and Warrington, 1984, 1987; Martin, 1996; Martin and Saffran, 1997; Gold and Kertesz, 2001; Bartha and Benke, 2003). Repetition in phonologically-impaired patients with left hemisphere involvement (e.g., CA) is generally reliant on lexical-semantic processing (McCarthy and Warrington, 1984, 1987; Martin and Saffran, 1997; Jefferies et al., 2007). The use of this alternative strategy increases the likelihood of producing word errors (formal paraphasias) and semantic errors particularly in highly demanding tasks such as immediate serial repetition of word lists and sentences and delayed repetition (Martin et al., 1994; Martin, 1996; Gold and Kertesz, 2001; Jefferies et al., 2006). Additionally, reliance on lexical-semantic processing in some conduction aphasic patients with severely abnormal phonological processing is manifested by “part of speech” effects (e.g., nouns are repeated better than verbs) and production of semantic paraphasias (“necklace” → *gold*) during single word repetition (deep dysphasia) (Michel and Andreewsky, 1983; Katz and Goodglass, 1990; Butterworth and Warrington, 1995; Martin, 1996; Martin et al., 1996; Ablinger et al., 2007; Jefferies et al., 2007). Such overreliance on lexical-semantic processing allows CA patients to excel in repetition tasks tapping these functions relative to other tasks taxing phonological processing. In this vein, patients with typical CA show better repetition of low-frequency words embedded as the last word in a sentence than when the same word is presented in isolation (McCarthy and Warrington, 1984). Abnormal performance in repeating meaningless word lists by conduction aphasics improves when the meaningfulness of lists is increased (McCarthy and Warrington, 1987) and these patients are also better able to repeat novel sentences which require access to meaning than over-learned idiomatic clichés (McCarthy and Warrington, 1984; Berthier, 1999). Finally, verbatim repetition of word lists and sentences poses serious difficulties to conduction aphasics due to their impaired capacity to hold the phonological trace in auditory-verbal short-term memory forcing them to process sentences by meaning and producing paraphrases of the target sentence during repetition (Saffran and Marin, 1975; Martin, 1993; Bartha and Benke, 2003).

Our patients repeated words more accurately than non-words and in one patient (JAM) stimulus length influenced more than frequency/imageability the dissociation between word and non-word repetition, whereas the reverse pattern was true for the other patient (AFL). Nevertheless, the occurrence of other abovementioned features of typical CA did not occur in all repetition tasks in our patients. Indeed, frequency/imageability, and grammatical class had no influence on single word repetition performance, although we acknowledge that in one such task (imageability/frequency) both patients obtained high scores that may have attenuated differences due to ceiling effects. This effect was not observed in JAM in the other task (grammatical class), however. Word pair repetition was moderately impaired and a marginal effect of frequency/imageability was only found in AFL. Moreover, patients produced more omissions and phonological errors than formal errors or word pair repetition and there were no semantic paraphasias, a pattern of performance that differs from the “lexical bias” (formal and semantic errors > phonological errors) reported in patients with typical CA and left hemisphere damage (Gold and Kertesz, 2001). Since word triplet repetition was extremely poor in both patients, we analyzed the accuracy of individual words on triplets. There was an influence of frequency in both patients who produced more correct items while repeating high-frequency than low-frequency lists. Moreover, they accurately repeated more individual words in triplets containing meaningful semantic information than in other conditions, thus implying that accurate repetition required semantic support. However, reliance on lexical-semantic processes could be deemed incomplete because both patients did not produce meaning-based paraphrases (e.g., “eat-delicious-apple” → *eat-juicy-fruit*) which is at variance to that frequently reported in patients with typical CA during repetition of two- and three-word lists (Gold and Kertesz, 2001; Berthier et al., 2012). Repetition of sentences from PALPA 12 was severely impaired in both patients and again paraphrases of target sentences were absent in AFL and JAM rarely produced ill-formed paraphrases in this task, novel sentences and clichés. Limited lexical-semantic access during word triplet and sentence repetition is in accord with findings from the two previous cases of crossed CA (Berndt et al., 1991; Berthier et al., 2011). Moreover, superior repetition of novel sentences over idiomatic clichés previously reported in typical CA patients (McCarthy and Warrington, 1984) reflecting overreliance on lexical-semantic processes was not observed in JAM (this test was not administered to AFL). Finally, it should be noted that JAM had more reliance on lexical-semantic processes in other output modalities (reading and spelling) (De-Torres et al., *in preparation*), a dissociation already reported in other patients with “deep” disorders (e.g., Miceli et al., 1994; Jefferies et al., 2007). Analysis of further cases is clearly needed to examine whether or not interactions between phonological and lexical-semantic systems in crossed CA are dysfunctional.

If one accept that JAM, AFL and the two previously published cases, EDE (Berndt et al., 1991) and JNR (Berthier et al., 2011) had limited access to meaning at least during sentence comprehension and repetition, the question arising now is which neural mechanisms are dysfunctional. Analysis of available brain images in these two previous cases and the outline of white matter tracts with the aid of a fiber tract atlas (Catani and Thiebaut de Schotten, 2012) in JAM and AFL and DTI analysis in JAM revealed that cortical and subcortical lesions unfailingly compromised the right dorsal (AF) and ventral auditory processing streams (IFOF) in all cases. DTI in JAM disclosed damage to the right long direct segment of the AF and IFOF with relative sparing of the anterior indirect and posterior segments, together with fully developed left AF and IFOF. Although DTI could not be performed in AFL (she was studied in 1997), her anatomical T_1_-weighted images were compared with a human atlas of fiber tract connections (Catani and Thiebaut de Schotten, 2012) and revealed compromise of AF and IFOF. The role of the dorsal language stream system (AF/SLF) is to monitor auditory-motor integration of speech by allowing a fast and automated preparation of copies of the perceived speech input (Saur et al., 2008; Peschke et al., 2009; Rijntjes et al., 2012). Some components of this long-distance bundle have also been linked to attention and short-term maintenance of phonological traces (Majerus, 2013). The ventral language pathways (inferior longitudinal fasciculus, IFOF and uncinate fasciculus) participate in comprehension by mapping sounds onto meaning (Saur et al., 2008; Peschke et al., 2009; Weiller et al., 2011; Cloutman, 2012) although the precise functional role of every tract is still controversial (Duffau et al., 2009; Harvey et al., 2013). These white matter bundles are engaged in different language functions (Hickok and Poeppel, 2004; Rolheiser et al., 2011; Weiller et al., 2011; Cloutman, 2012; Friederici and Gierhan, 2013) although they interact in a synergistic way (Rolheiser et al., 2011; Cloutman, 2012; Majerus et al., 2012; Majerus, 2013), so that phonological sequencing and articulation from the dorsal stream operate in concert with the semantic information from the ventral stream to guarantee efficient production and comprehension of language (Turken and Dronkers, 2011; Cloutman, 2012; Friederici and Gierhan, 2013; Rijntjes et al., 2012). Therefore, impaired sentence comprehension and repetition of non-words, word lists and sentences in JAM and AFL may be ascribed to the simultaneous damage to the ventral (AF) and dorsal (IFOF) streams.

JAM, AFL and the two previous cases, EDE and JNR (Berndt et al., 1991; Berthier et al., 2011) also had variable cortical involvement which definitely contributed to the observed deficits. Right temporo-parietal involvement (large in EDE and JRN and mild to moderate in JAM and AFL) was heterogeneous but consistently involved the right ventral temporal cortex encompassing the temporal stem and its adjoining auditory and visual white matter tracts. Comprehension deficits in acute (Naeser et al., 1982; Kümmerer et al., 2013) and chronic aphasia (Alexander et al., 1989b; Sharp et al., 2004) have been correlated with dysfunction of ventral temporal cortex and interruption of long-distance association (ventral stream—IFOF) and commissural (anterior commissure) cortico-cortical pathways (Sharp et al., 2004; Warren et al., 2009; Turken and Dronkers, 2011; Weiller et al., 2011; Cloutman, 2012; Friederici and Gierhan, 2013). Functional neuroimaging and brain stimulation studies also found that the basal temporal cortex, frontal operculum and the ventral stream are strongly engaged in lexical-semantic and syntactic processing (Nobre et al., 1994; Sharp et al., 2004; Warren et al., 2009; Rolheiser et al., 2011; Friederici and Gierhan, 2013; Koubeissi et al., 2012; Weiller et al., 2011). In consonance with these data, our patients and the two previously published cases (Berndt et al., 1991; Berthier et al., 2011) had auditory and written comprehension preserved for single words but not for sentences presented in these input modalities. The basal ganglia components of the lesions in our patients involved the anterior commissure (Warren et al., 2009; Catani and Thiebaut de Schotten, 2012) and probably interrupted functional connectivity between homologous regions of the anterior and medial temporal cortex, thus preventing access to meaning in the left temporal cortex during sentence comprehension/production (Umeoka et al., 2009; Warren et al., 2009). In addition, tissue damage to the right basal temporal cortex is highly likely to disrupt its reciprocal connectivity with the posterior-superior temporal gyrus further hampering phonological processing (Ishitobi et al., 2000; Koubeissi et al., 2012). Therefore, it seems that damage to these structures might have impeded in our patients a compensatory recruitment of the lexical-semantic system in the service of repetition as in usually observed in patients with chronic CA and left hemisphere damage.

### Limitations

One important shortcoming of our study is that formal language evaluations could be performed only in the chronic period. This precluded determining whether some functions were spared (e.g., single word comprehension) because they were unaffected by tissue damage or whether they were abnormal in the early stages and recovered later on reflecting the action of compensatory mechanisms associated with either brain reparation or the recruitment of alternative brain areas. Future studies in aphasic patients like the ones described here should be longitudinal, initiated soon after brain damage, and complemented with multimodal imaging (e.g., fMRI, arterial spin labeling, positron emission tomography) to evaluate dissociation of language functions and also to rule out remote effects in the contralateral hemisphere.

## Concluding remarks

In conclusion, our findings reveal that patients with crossed CA and right striatal/capsular lesions extending inferiorly to the temporal stem and IFOF and superiorly to the AF and white matter beneath the supramarginal gyrus may show limited access to lexical-semantic information during word list and sentence repetition. Interruption of the long direct segment of the right AF might account for the abnormal performance in word and non-word repetition. Damage to the right ventral stream (IFOF) running between the insular cortex and putamen might be responsible from the impairment of the lexical-semantic and syntactic processing necessary for accurate sentence comprehension and repetition. In addition, the involvement of the right basal temporal cortex (temporal stem, basal language area) may have severed commissural pathways (anterior commissure) disrupting functional connectivity with its homologous counterpart further limiting the access to meaning during sentence comprehension/production (Umeoka et al., 2009; Warren et al., 2009) and also with the posterior-superior temporal gyrus disturbing phonological processing (Ishitobi et al., 2000; Koubeissi et al., 2012). Further analysis of individuals with right hemisphere language dominance is needed to enhance our understanding on the role of white matter tracts in language repetition.

### Conflict of interest statement

The authors declare that the research was conducted in the absence of any commercial or financial relationships that could be construed as a potential conflict of interest.

## Footnotes

^1^Conventional speech-language therapy in both patients followed a syndrome-specific standard approach. The therapeutic repertoire ranged from exercises involving naming, repetition, sentence completion, following commands, spoken object-picture matching, and conversations on topics of the patients' own choice (see Pulvermüller et al., 2001; Basso, 2003; Basso et al., 2013).
